# Hybridization Between Yuccas From Baja California: Genomic and Environmental Patterns

**DOI:** 10.3389/fpls.2020.00685

**Published:** 2020-05-28

**Authors:** Maria Clara Arteaga, Rafael Bello-Bedoy, Jaime Gasca-Pineda

**Affiliations:** ^1^Departamento de Biología de la Conservación, Centro de Investigación Científica y de Educación Superior de Ensenada (CICESE), Ensenada, Mexico; ^2^Facultad de Ciencias, Universidad Autónoma de Baja California, Ensenada, Mexico; ^3^Unidad de Biotecnología y Prototipos (UBIPRO), Facultad de Estudios Superiores Iztacala, Universidad Nacional Autónoma de México, Mexico City, Mexico

**Keywords:** endemism, climatic change, hybridization, mutualism, *Yucca valida*, *Yucca capensis*

## Abstract

Hybridization can occur when two geographically isolated species are reproductively compatible and have come into sympatry due to range shifts. Yucca and yucca moths exhibit obligate pollination mutualism; yucca moths are responsible for the gene flow mediated by pollen among yucca populations. In the Baja California Peninsula, there are two yucca sister species, *Y. capensis* and *Y. valida*, that have coevolved with the same pollinator, *Tegeticula baja*. Both yucca species are endemic to the peninsula, and their current distributions are allopatric. Based on their morphological characteristics, it has been suggested that some plants growing in the southern part of the Magdalena flatland, a spatially disjunct part of *Yucca valida*’s range, have hybrid origins. We conducted genomic and climatic analyses of the two yucca species as well as the putative hybrid populations. We genotyped 3,423 single nucleotide polymorphisms in 120 individuals sampled from 35 localities. We applied Bayesian tests and geographic cline analyses to the genomic data. Using climatic information from the occurrence sites, we projected species distribution models in different periods to assess changes in the distributional range, and we performed a statistical test to define the niche divergence between the paternal species and the putative hybrid populations. Structure analysis revealed mixed ancestry in the genome of hybrid populations, and the Bayesian models supported a scenario of post-divergence gene flow between the yucca species. Our species distribution models reveal that the geographical ranges of the parental species overlapped mainly during the Last Glacial Maximum, which could facilitate genetic admixture between those species. Finally, we found that most of the assessed environmental axes between the parents and hybrid populations are divergent, indicating that the climatic niche of the hybrid populations is shifting from that of the populations’ progenitors. Our results show that the populations in the southern part of the Magdalena flatland are the result of combination of the genetic components of two species. Hybrid individuals with this novel genomic combination arose in a different habitat than their parental species, and they exhibit ecological divergence, which contributes to reproductive isolation through spatial and temporal barriers.

## Introduction

Hybridization, which is defined as the reproduction of members of genetically distinct populations that produce offspring of mixed ancestry (Barton and Hewitt, 1985), plays a pivotal role in evolution and diversification (Rieseberg, 1997; Taylor and Larson, 2019). There is ample evidence of hybridization in plants some of which has lead to the subsequent diversification of plant lineages (Arnold, 2015). In a spatial context, hybridization can occur when two geographically isolated species are reproductively compatible and have come into sympatry due to range shifts. Over time, climates have changed and species have responded by expanding or contracting their geographic distributions, causing some of them to become sympatric over parts of their ranges with the potential to interbreed during some periods of their evolutionary histories (e.g., Zinner et al., 2009; Cahill et al., 2018). Commonly, the biogeographic distributions of parents species are allopatric with respect to their hybrid derivatives (Gross and Rieseberg, 2004), which can happen when the environmental conditions of the area occupied by hybrids do not favor the establishment of parental species. Interspecific gene flow can generate new combinations of alleles, which create phenotypes that are able to explore novel habitats. The colonization of new habitats may allow hybrids to avoid competition with their parental species, and reproductive isolation can be achieved through ecological divergence and/or geographical isolation (e.g., Brochmann et al., 2000; Abbott et al., 2013).

Closely related species often hybridize (Abbott et al., 2013) if they share ecological attributes. For example, obligate mutualisms between flowering plants and their pollinators can favor genetic exchange and hybridization (Arnold, 2015), when pollinators do not differentiate between host species and plant reproductive barriers are incomplete (e.g., Leebens-Mack and Pellmyr, 2004; Kawakita and Kato, 2006). This has been the case in the obligate pollination mutualism between yuccas and yucca moths. The major reproductive barriers to gene flow among yucca species are spatial and temporal isolation and pollinator preference, and when these barriers collapse, hybridization can take place (Lenz and Hanson, 2000). There are several examples in which hybridization result from heterospecific pollination among sympatric yucca species (Miles, 1983; Leebens-Mack et al., 1998; Lenz and Hanson, 2000; Rentsch and Leebens-Mack, 2012; Starr et al., 2013). Within this genus, the hybridization has generated high morphological and genomic variation, including hybrids that coexist with their parents (Miles, 1983; Leebens-Mack et al., 1998; Lenz and Hanson, 2000; Starr et al., 2013; Royer et al., 2016) as well as new species (Rentsch and Leebens-Mack, 2012).

The two sister species *Yucca valida* Brandegee and *Yucca capensis* Lenz are long-lived monocot trees endemic to the Baja California Peninsula, located in northwestern México. Those species currently exhibit an allopatric distribution and grow under different environmental conditions. Populations of *Y. valida* grow in arid ecosystems from the Central Desert (30°N) to the Magdalena flatland, and this species show high density of individuals across the landscape (Turner et al., 1995). Plants of *Y. valida* flower between April and July (Turner et al., 1995; personal observation). In turn, populations of *Y. capensis* mainly grow in a remnant of tropical deciduous forest along the mountains located in the southern part of the Baja California Peninsula (Lenz, 1998; De la Luz et al., 2012), and consist of groups of less than 15 individuals separated by considerable distances (Lenz, 1998; Arteaga et al., 2015). Flowering of *Y. capensis*, in part, is determined by rainfall, with the boom during September and October (Lenz, 1998; Arteaga et al., 2015). These two sister Yucca species are pollinated by the same yucca moth, *Tegeticula baja* Pellmyr (Pellmyr et al., 2007, 2008). Briefly, after emerging from the cocoon, the female moth uses specialized mouthparts to collect pollen from yucca flowers and carry it to other flowers. She cuts into the floral ovary with her ovipositor and injects eggs. Then, she walks up to the stigma and actively deposits the pollen to fertilize the flower. The fruit develops, and her progeny feed on a small portion of the seeds (Pellmyr, 2003).

Until the mid-1990s, *Y. valida* and *Y. capensis* populations were considered a single species, *Y. valida*, and the large phenotypic variation was attributed to the ample variation in climatic conditions experienced by members of the species inhabiting the peninsula (Turner et al., 1995). However, based on their morphological characteristics, populations located in the tropical deciduous forest were described as a new species, named *Y. capensis* (Lenz, 1998). Moreover, the yucca plants that grow in the southern part of the Magdalena flatland (23.5°–24.5°N), show phenotypic traits of leaves and stems that resemble those of *Y. valida* and *Y. capensis*, leading to the suggestion that those populations originated through hybridization of both endemic yuccas (Lenz, 1998). Morphological inspection of those plants lends partial support to that hypothesis (personal observation), but the allopatric distribution of *Y. valida* and *Y capensis* makes it difficult to infer whether those plants are hybrids. However, it is possible that secondary contact between endemic yuccas occurred in the past, and the arrangement of new genetic combinations allowed putative hybrids to occupy environmental conditions that were unexplored by the parental species (Arnold, 2015).

In this study, we integrated genomic and climatic analyses to assess whether populations located in the southern part of the Magdalena flatland are *Y. valida* × *Y. capensis* hybrids. We sampled individuals across the geographical distribution of both endemic species as well as the putative hybrid populations, and we genotyped a total of 120 plants derived from 35 localities within the distribution ranges. We applied Bayesian tests and geographic cline analyses to the genomic data, and using climatic data from the occurrence sites, we did species distribution models (SDMs) and a statistical test to define the niche divergence among taxa. Our specific goals were to examine (i) whether populations located in the southern part of the Magdalena flatland originated through hybridization between *Y. capensis* and *Y. valida* and their level of admixture; (ii) whether Quaternary climate change influenced a shift in species distribution that favored hybridization and the establishment of hybrid descendants; and (iii) whether there is ecological divergence among putative hybrid populations and endemic yucca species.

## Materials and Methods

### Sampling Area, DNA Extraction, and Genotyping

**FIGURE 1:**
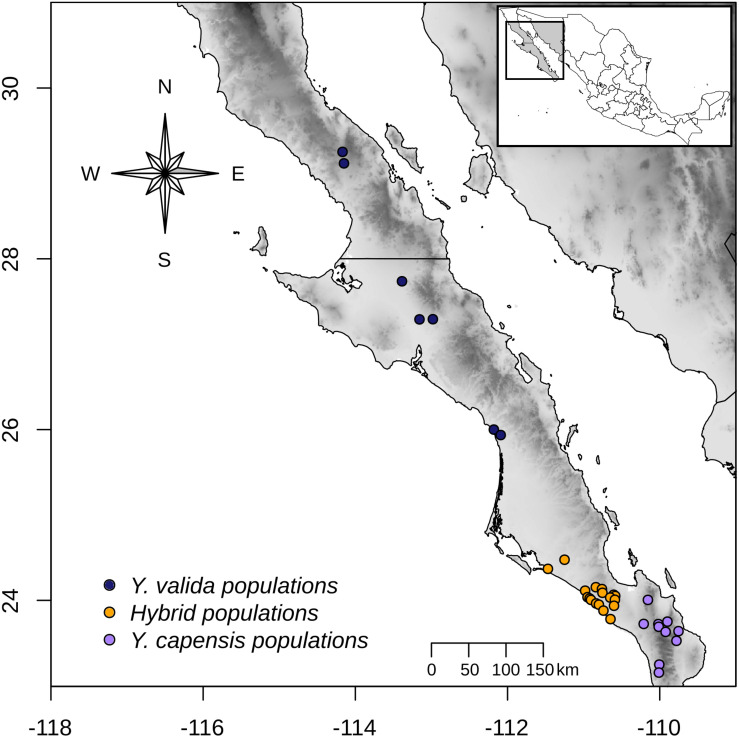
Sampling locations of *Y. valida* (blue), *Y. capensis* (purple), and the putative hybrid populations (orange) in the Baja California Peninsula.

Across a transect of 800 km (from latitude 23° to latitude 26° N), we collected 10 g of fresh leaf tissue from 120 individuals from 35 localities (Figure 1). Using this sampling scheme, we covered the geographical range of *Y. valida* Brandegee and *Y. capensis* Lenz and the known range of the putative hybrid populations, which were located in the southern part of the Magdalena flatland (23.5°–24.5°N). Based in the geographical locations where we collected the individuals, we assigned them to a putative species or to the hybrid. We dried the tissues using silica gel for preservation. We extracted total genomic DNA from 100 mg of disrupted lyophilized leaf tissue using the DNeasy Plant Mini Kit (Qiagen, Hilden, Germany). We assessed the quantity and integrity of DNA using 1.5% GelRed stained agarose gels and a NanoDrop spectrophotometer (Thermo Fisher Scientific, Waltham, MA, United States).

We used the genomic DNA to generate nextRAD libraries, following previously described strategies (SNPsaurus, LLC; Russello et al., 2015). This method uses selective PCR primers to amplify genomic loci consistently among samples. Genomic DNA was first fragmented with Nextera reagent (Illumina, Inc., San Diego, CA, United States), which ligates short adapter sequences to the ends of the fragments. The Nextera reaction was scaled to fragment 25 ng of genomic DNA, and 50 ng of genomic DNA was used as an input to compensate for the amount of degraded DNA in the samples and to increase fragment sizes. Then, fragmented DNA was amplified for 27 cycles at 74°C, with one of the primers matching the adapter and nine nucleotides extending into the genomic DNA with the selective sequence GTGTAGAGCC. Thus, only fragments starting with a sequence that can be hybridized by the selective sequence of the primer would be efficiently amplified. The nextRAD libraries were sequenced on a HiSeq 4000 with one lane of 150 bp reads (University of Oregon). Raw reads are available in NCBI under the SRA numbers: SRR11514779, SRR11514778, and SRR11514777.

### Raw Data Processing and Filtering

We checked the quality of raw sequence reads using FASTQC v0.11.5 (Andrews, 2010) with a threshold of *q* > 29. We removed the adapter sequences and low-quality reads using the *bbduk* module of the BBTools v37.87 software package^1^. All reads were end-trimmed to a length of 99 pb. Afterwards, we rechecked the reads’ quality with FASTQC.

We built loci using a *de novo* assembly module of the Stacks v2.4 pipeline (Catchen et al., 2011, 2013). To obtain the optimal set of parameters, we used the procedure proposed by Paris et al. (2017). This procedure involves iterating different ranges of values for the parameters *m* (the minimum number of identical reads required to create a stack), *M* (the number of mismatches allowed between loci on a single individual), and *n* (the number of mismatches allowed between loci when building the catalog). We plotted and evaluated the resultant metrics to select the optimal set of parameters that maximizes the amount of reliable information. The parameter *r* (the minimum percentage of individuals required to process a locus) was fixed at 0.80, as recommended by Paris et al. (2017). The final set of parameters were *m* = 3, *M* = 3, and *n* = 4. When a locus showed more than one segregating site, one variant was randomly sampled from that locus to ensure that the loci were mostly independent. We discarded single-nucleotide polymorphisms (SNPs) with a minimum allele frequency (MAF) smaller than 0.05. Then, we used plink v1.07 (Purcell et al., 2007) to exclude individuals with more than 15% missing loci and loci that failed the Hardy-Weinberg equilibrium (HWE) test at *p* < 0.05. Moreover, to assess the degree of linkage disequilibrium among selected loci, we estimated correlation *r*^2^ index and we considered those loci with *r*^2^ < 0.8. Regarding the ploidy of these species, both endemic yuccas as well as the hybrid are diploids.

### Genomic Diversity and Structure

We estimated observed and expected heterozygosities as well as inbreeding coefficients (H_E_, H_O_, F_IS_) using the R v3.41 R Core Team 2017 packages “adegenet” v2.1 (Jombart and Ahmed, 2011), “hierfstat” v0.4-22 (Goudet and Jombart, 2015), and Genepop v4.6 (Rousset, 2008). We also estimated pairwise differentiation (F_ST_) among the species and putative hybrid populations, and we evaluated the statistical significance using the gstat. randtest function of the “hierfstat” package in 10,000 simulations. As a first approach to explore the grouping of individuals as a function of their allele composition, we performed a principal component analysis (PCA) at the individual level using the “adegenet” package.

To evaluate the genetic structure of the taxa, we used two approaches. First, we ran Structure v2.3.4 (Pritchard et al., 2000; Falush et al., 2003) using the admixture model with correlated allele frequencies and a burnin period of 150,000 steps and 500,000 iterations. We tested one to eight clusters (*K*), performing 15 iterations each. The software was executed in parallel using the StrAuto v1.0 script (Chhatre and Emerson, 2017). To determine the most likely value of *K*, we performed the Evanno test (Evanno et al., 2005), which was implemented in Structure Harvester v0.6.94 (Earl, 2012). We also plotted the likelihood values for each *K*-value to identify the highest value of *K* with the lowest variance. Additionally, we used the method for discriminant analysis of principal components (DAPC) proposed by Jombart et al. (2010). This method relies on the use of multivariate individual information (in this case, genotypes) using discriminant functions, which optimize the variance between groups and minimize the variance within clusters. To select the number of retained components as well as the number of discriminant functions, we performed a cross-validation test. The number of possible clusters was assessed using the Bayesian Information Criterion (BIC), which was run 10 times to ensure the stability of the results. DAPC analysis was carried out using the “adegenet” package.

### Evaluation of Historical Scenarios Using ABC Analysis

To approximate the evolutionary history of the endemic yuccas and putative hybrid populations, we used DIY-ABC v2.1 (Cornuet et al., 2014). We tested five scenarios related to the origin of hybrid plants located in the Magdalena flatland (Supplementary Figure S1). Scenarios A and B proposed that the putative hybrid populations originated from *Y. valida* and *Y. capensis*, respectively. Scenario C proposed that the hybrid populations diverged before the divergence of *Y. valida* and *Y capensis*. Scenario D proposed that *Y. valida* and *Y. capensis* diverged and the putative hybrid populations are a result of mixture of the two genetic pools. Scenario E proposed that the three taxa diverged simultaneously in the past, and they have independent demographic histories. We ran 2 × 10^6^ simulations per scenario, as recommended by the software. We divided the entire data set into three sets and ran them independently, resulting in a total of 6 × 10^6^ simulations per scenario.

We used the proportion of monomorphic loci, the mean genetic diversity, and the variance across polymorphic loci, as well as the mean genetic diversity across all loci as “single sample statistics.” For the “two-sample statistics,” we used the proportion of loci with null F_ST_ and Nei distances, the mean and variance across loci with non-null F_ST_ and Nei distances, and the mean and variance across loci of F_ST_ and Nei distances. In addition, we used the mean of admixture across all loci, the mean and variance of non-null admixture estimates, and the proportion of loci with null admixture as the “three sample statistics. To check if the combination of scenarios produced simulated data close to the actual data set (pre-evaluation), we implemented a PCA using 10,000 simulations. Then, we estimated the posterior probabilities of different scenarios by fitting a multinomial logistic regression using the 1% of simulated dataset closest to the observed data, followed by linear discriminant analysis. The goodness of fit of the scenario (“Model checking” option) was carried out by simulating 1,000 pseudo-observed data sets with the posterior model’s distribution combination with parameter values drawn from 1,000 sets of the posterior sample. The summary statistics of the actual data were ranked with the posterior distribution of the scenarios’ summary statistics. Finally, we estimated type-I and type-II errors for all scenarios implementing the “evaluate the confidence in scenario choice” option.

### Geographic Cline Analyses

We performed sigmoid cline analyses of the allele frequencies for each SNP and the admixture index to identify genomic signals of hybridization across the geographical distribution of the yucca populations (hybrid zone, Abbott et al., 2013). We used the population average of the individual assignment probabilities (*q*-value) obtained with Structure for *K* = 2 as admixture index of the two putative parental species, *Y. valida*, and *Y. capensis.* Clines were fitted using the R package “hzar” v0.2-5 (Derryberry et al., 2014). First, we calculated the geographic distance between all sampling localities against the northernmost sampled locality using the function *pointDistance* in the R package “raster” v2.6-7 (Hijmans et al., 2017). We tested three models: (i) pmin/pmax set to the observed values without fitted exponential decay curves (i.e., tails; model I), (ii) estimated pmin/pmax with no fitted tails (model II), and (iii) estimated pmin/pmax and both tails fitted (model III). Models were evaluated based on the Akaike Information Criterion corrected for small sample size (AICc). The model with the lowest AICc was considered to have the best fit.

### Environmental Data, Testing for Niche Divergence, and Species Distribution Modeling

To test for niche divergence, we employed the multivariate method introduced by McCormack et al. (2010), which compares niche divergence to a null hypothesis of divergence in available background environments on several orthogonal axes of environmental space. The method also uses PCA to reduce the raw GIS data into a smaller, uncorrelated set of axes. The general idea behind McCormack et al. (2010) method is that a pattern of divergence in GIS data can be attributed to either meaningful niche divergence between species or a strong spatial autocorrelation between GIS data. Therefore, a robust test of niche divergence or conservatism must compare niche divergence between taxa to the baseline levels of divergence drawn from the background of the available habitat within each taxon’s geographic range. The null hypothesis is rejected when niches are more similar (niche conservatism) or more different (niche divergence) between taxa than the null model of background divergence. If the null hypothesis is not rejected, this does not mean that there is no niche divergence between the taxa, but that divergence between taxa (whether meaningful or due to spatial autocorrelation) is plausible.

To conduct the multivariate test for niche divergence, we used 19 BioClim layers (Bio1-Bio19)^2^ that describe aspects of temperature, precipitation, and seasonality as well as potentially biologically limiting extremes of these variables. BioClim layers have a resolution of 1 km^2^. To describe the climatic niche used by each taxon, we extracted raw data from 428 unique records (localities), 385 of which were from *Y. valida*, 18 of which were from *Y. capensis*, and 25 of which were from the putative hybrid populations. The latitude and longitude coordinates for individuals sampled and observed in the field were obtained from a global positioning system tracker. To generate the background predictions for each taxon, we developed a distribution polygon by drawing 5 km circles around each individual and merged them to obtain a continuous area. Then, we placed 2,000 random points inside each polygon and extracted raw data from them. We used the R packages “sp” (Pebesma and Bivand, 2005) and “dismo” (Hijmans et al., 2017). Because we were interested in determining whether putative hybrid populations ecologically diverge from their parents, we performed two pairwise analyses. One was conducted between *Y. valida* and putative hybrid populations, and the other was conducted between *Y. capensis* and putative hybrid populations. For each pairwise analysis, we joined the climatic data from records and background polygons of the two taxa, and we conducted a PCA. We extracted the first three principal component (niche) axes for further consideration since they comprised the bulk of the variation and were readily interpretable (see the Results section). Niche divergence or conservatism was evaluated on each niche axis by comparing the observed difference between the means for each yucca species and the putative hybrid populations on that axis to the mean difference in their background environments on the same axis. A null distribution of background divergence was created by recalculating the background divergence score over 1,000 jackknife replicates with 75% replacement. Significance for rejecting the null was evaluated at the 95% level. All analyses were conducted using Stata v10.

To evaluate the influence of past environmental conditions on possible distribution overlap of the taxa, we built species distribution models (SDMs) using BioClim layers^2^ (Hijmans et al., 2005) for the present, mid-Holocene (MIROC-ESM; Watanabe et al., 2011), Last Glacial Maximum (MIROC-ESM; Watanabe et al., 2011), and Last Interglacial (Otto-Bliesner et al., 2006) periods. To avoid possible bias due to highly correlated variables, we extracted data from the 19 BioClim layers and conducted paired Pearson correlation tests with a threshold of >0.75. From each pair of correlated variables, we selected the variable that had more than one significant correlation with another variable. Additionally, we estimated the variance inflation factor using the vifcor function of the R package “usdm” (Naimi et al., 2014). Using both criteria, we retained twelve bioclimatic variables: *BIO1* (annual mean temperature), *BIO2* (mean diurnal range), *BIO4* (temperature seasonality), *BIO5* (maximum temperature of warmest month), *BIO8* (mean temperature of wettest quarter), *BIO9* (mean temperature of driest quarter), *BIO12* (annual precipitation), *BIO13* (precipitation of wettest month), *BIO14* (precipitation of driest month), *BIO15* (precipitation seasonality), *BIO18* (precipitation of warmest quarter), and *BIO19* (precipitation of coldest quarter).

We constructed the final models based on an ensemble of forecasting models using the committee-averaging criteria (Araújo and New, 2007) in the R package “biomod2” v3.1 (Thuiller et al., 2019). The ensemble uses four algorithms: (1) a generalized linear model (McCullagh and Nelder, 1989), (2) a generalized boosted model (Friedman, 1991), (3) MaxEnt (Phillips et al., 2006), and (4) random forests (Breiman, 2001). To reduce possible autocorrelation bias due to local overrepresentation of records, we generated a grid of 1/10 degree cells for each taxon and then selected one point at random from each cell using the “raster” package. Two independent pseudo-absence sets of 5,000 points were generated at random, and the species records were split (70% for model training and 30% for evaluation of the model’s performance). With the 70–30 criterion, we ran five random replicates for all models. We assessed the models’ performance using the area under the receiver operating characteristic curve (AUC; Swets, 1988) and the true skill statistic (TSS; Allouche et al., 2006). Ensembles were restricted to models with AUC > 0.9 and TSS > 0.8, and we transformed them into binary data using the evaluation metrics and thresholds obtained by TSS evaluation.

## Results

### Genomic Diversity and Structure

After quality checking and filtering, the final data set consisted of 3,423 biallelic loci from 103 individuals from 35 localities. The highest value of linkage disequilibrium was *r*^2^ = 0.72, and more than 97.5% of paired values were lower than *r*^2^ = 0.5, so we keep all loci for further analyses. The two endemic yuccas and hybrid populations had an overall genetic diversity of H_E_ = 0.2826 and H_O_ = 0.1255 for the 3,423 analyzed SNPs. The three taxa show differences in genetic diversity, and the putative hybrid populations had the highest diversity. We also found a significant deficiency of heterozygotes in the three taxa (Table 1). In addition, fixed loci were common in *Y. valida* Brandegee and *Y. capensis* Lenz, and polymorphic loci were mostly found in hybrid populations (Supplementary Figure S2). Finally, the hybrid populations had only 28 private alleles, while we detected 40 private alleles in *Y. valida*, and 78 in *Y. capensis*.

**TABLE 1:** Number of localities and individuals sampled per taxon, and summary statistics of genetic diversity (H_E_, expected heterozygosities; H_O_, observed heterozygosities; and F_IS_, inbreeding coefficient).

Taxa	Localities (Ind)	H_E_	H_O_	F_IS_
*Y. valida*	7 (35)	0.1736	0.1119	0.3522
Hybrid pops.	18 (43)	0.2606	0.161	0.3802
*Y. capensis*	10 (25)	0.2496	0.1036	0.5763
Overall	35 (103)	0.2826	0.1255	0.5338

**FIGURE 2:**
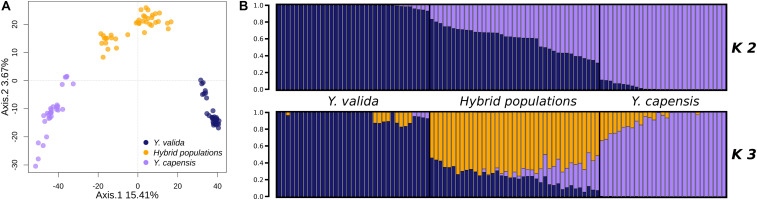
**(A)** Principal component analysis of the 3,423 SNPs obtained for *Y. valida*, *Y. capensis*, and the hybrid populations. Dots represent individuals. **(B)** Bayesian cluster analysis of *Y. valida*, *Y. capensis*, and the putative hybrid individuals using Structure (*K* = 2 and *K* = 3). Genetic clusters are coded with different colors. Individuals were ordered according to the latitude of their locality of origin.

The overall value of genomic differentiation among the three taxa was F_ST_ = 0.1939. While the level of structure between the endemic species (*Y. valida* and *Y. capensis*) was high (F_ST_ = 0.2538), the values of the paired comparisons with hybrid populations were lower (*Y. valida*–hybrid populations: F_ST_ = 0.099; *Y. capensis*–hybrid populations: F_ST_ = 0.102). Moreover, the PCA performed at the individual level showed clear separation of individuals of different yucca populations (Figure 2). The clustering analysis, which was performed with Structure using the Evanno test (Evanno et al., 2005), suggested the existence of two groups (*K* = 2; Figure 2). Close inspection of the ln (P) plots showed that individuals with higher assignment probabilities correspond to the localities of *Y. valida* (*q* ≥ 0.930) or *Y. capensis* (*q* ≥ 0.888) and all individuals with high levels of admixture belonged to the locality of the hybrid populations. Following the PCA analysis, DAPC revealed three genetic groups separating all individuals into their respective taxon (Supplementary Figure S3). This result was expected, as multivariate methods optimize the variance between groups without *a priori* assumptions, while Structure searches for the optimal grouping searching a Hardy-Weinberg equilibrium. Based on this, we included the barplot values for *K* = 3 (Figure 2), which allowed us to determine that the third genetic group predominates in individuals from the hybrid populations.

### Evaluation of Historical Scenarios and Geographic Cline Analyses

**FIGURE 3:**
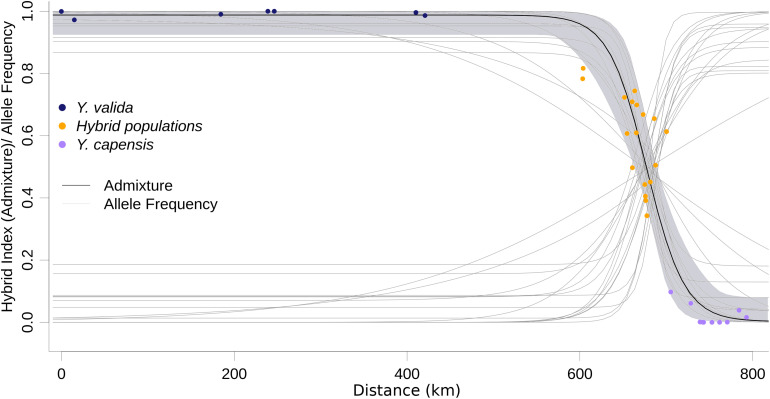
Maximum likelihood geographic cline for the hybrid index. Dots represent the 35 localities, and the shaded area represents the 95% interval of the fitted model. Light gray lines represent the clines for the 174 diagnostic loci.

ABC modeling of historical scenarios provided unambiguous support for scenario D (Supplementary Figure S1), which suggests that hybrid populations are the result of a mixture of the previously diverged species *Y. valida* and *Y. capensis*. The cline of the admixture accurately described the geographic transition between *Y. valida* and *Y. capensis* (Figure 3). We found 176 diagnostic loci (i.e., loci that fit the sigmoidal cline model), 30 of which exhibited coincidence with the center of the admixture cline.

### Testing for Niche Divergence and Species Distribution Modeling

For comparison between *Y. valida* and the hybrid populations, the PCA identified three main niche axes that explained 82% of the variation. The first niche axis was associated with mean temperatures and rain seasonality; the second was associated with the annual range (variation) and seasonality of temperature and the lowest temperatures; and the third was associated with the highest temperature and precipitation and annual rain. The results of the niche divergence test showed evidence of conservatism on the first niche axis and divergence on the second and third axes (Table 2). Both taxa have conserved niches related with mean temperatures and rain seasonality (e.g., deserts). However, they diverge in terms of extreme temperature and rain values as well as the range of variation in temperature.

**TABLE 2:** Tests of niche divergence and conservatism.

	PC1	PC2	PC3
***Y. valida*-Hybrid pops.**			
Observed difference	4.3165*	1.367*	1.0301*
Null distribution	4.4744–4.6540	0.7710–1.0356	0.5880–0.7087
% variance explained	43%	26%	13%
***Y. capensis*-Hybrid pops.**			
Observed difference	4.4887	2.9678*	0.4564*
Null distribution	4.3197–4.5314	2.3443–2.4765	0.0520–0.1471
% variance explained	60%	20%	11%

Observed differences in the climatic niche of the hybrid populations and parental species on each PC axis are compared to the middle 95th percentile of the null distribution of the differences between their environmental backgrounds. Bold values indicate niche divergence. *Significance level, p < 0.05.

Between *Y. capensis* and the hybrid populations, PCA indicated that three main niche axes explained 92.3% of the variation. The first niche axis was associated with annual precipitation and extreme levels of rain; the second was associated with temperature and rain seasonality; and the third was associated with the minimum temperature of the coldest month. The results of the test indicate divergence on the second and third axes. The first niche axis did not significantly differ from the null expectation of background divergence (Table 2). Both taxa have divergent niches related to rain and temperature seasonality and the lowest temperatures.

**FIGURE 4:**
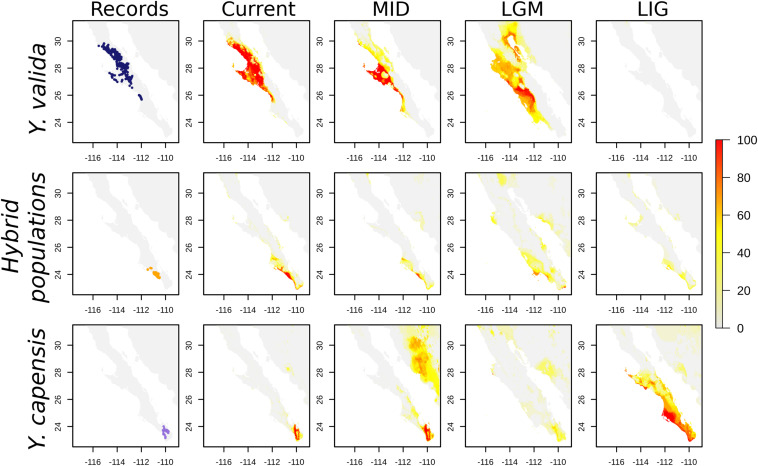
Georeferenced records and Species distribution models (SDMs) for *Y. valida*, *Y. capensis*, and the hybrid populations. The SDMs were generated for current conditions, the mid-Holocene (MID ∼6,000 years), the Last Glacial Maximum (LGM ∼21,000 years), and the Last Interglacial period (LIG ∼120,000 years). The color represents the habitat suitability values.

Finally, the SDMs indicate that the current distribution of both endemic yucca species does not overlap (Figure 4). Nevertheless, there are interesting results for past scenarios. The distribution of the suitable habitat for *Y. valida* reached lower latitudes during the mid-Holocene and LGM periods, but apparently, there was not suitable habitat in the center and south of the peninsula for the species during the Last Interglacial period. Although *Y. capensis* had the most restricted distribution in the present period, its suitable habitat was more widely distributed in the past, reaching the middle of the peninsula in the LIG period (around 28°N). Thus, the potential distribution of both endemic species overlapped in the past, mainly around latitudes of 25.5–23.5°N (Figure 4), which exhibit environmental suitability for both yucca species and correspond to the area currently occupied by the hybrid populations. A higher level of habitat suitability for the hybrid populations in the present period was found at latitudes of 25–22.8°N in a larger area than where they are currently observed (Figure 4).

## Discussion

Species geographic ranges are dynamic over evolutionary time. The large degree of allopatry among sibling species of yuccas and moth taxa suggest that diversification inside each group has been driven by geographical isolation rather than by reproductive isolation (Althoff et al., 2012). Thus, when two closely related species have come into sympatry due to range shifts, they could interbreed if their pollinators are not highly specialized. Our genomic data and Species Distribution Models support this scenario to endemic yuccas of the Baja California Peninsula. We confirmed the hybrid origin of yucca populations located in the southern part of the Magdalena flatland in the Baja California Peninsula. Climatic changes in the past probably caused geographic overlap of the distribution areas of *Y. capensis* Lenz and *Y. valida* Brandegee, which could facilitate genetic admixture between those species. Environmental analyses show that the hybrid populations are distributed in areas that are slightly different from those of the parental species, which can promote reproductive isolation.

### Genomic Patterns and Probable Historical Scenarios of Hybridization

One of the goals of this study was to assess whether populations located in the southern part of the Magdalena flatland have hybrid origins due to genetic exchange between *Y. valida* and *Y. capensis*. We found a clear grouping of two clusters corresponding to the two parental species and a third cluster that corresponded to individuals from the hybrid populations (Figure 2). In addition, those populations show the highest levels of genomic admixture compared to the level of admixture observed in individuals from the parental species. Although mixed ancestry in the genome of taxa is an important indicator of hybridization, it can be difficult to distinguish from ancestral polymorphism or continued gene exchange (Abbott et al., 2013). For this reason, we used alternative scenarios (ABC models) to test the most probable history that explains the data. We found the highest support for the model of a hybrid origin of these populations. Our results indicate that populations located in the southern part of the Magdalena flatland originated of hybridization between *Y. capensis* and *Y. valida.*

The occurrence of hybridization has been recorded at least four times among *Yucca* species, and it occurs in zones of host sympatry where yucca moths play an active role in the movement of heterospecific pollen between parental species (Miles, 1983; Lenz and Hanson, 2000; Rentsch and Leebens-Mack, 2012; Starr et al., 2013; Royer et al., 2016). Because yucca moths move pollen across short distances (Marr et al., 2000; Powell, 2013), heterospecific pollination could occur in places where parental species coexist, given that flowering phenologies overlap. The endemic species, *Y. valida* and *Y. capensis*, are pollinated by the same yucca moth species (Pellmyr et al., 2007), which could favor pollen flow between those hosts. However, in the current landscape, they have an allopatric distribution, suggesting that the hybridization events occurred in the past. Our SDMs reveal that the potential geographic distribution of the parental species changed since the Last Interglacial period and that their ranges show overlap mainly during the Last Glacial Maximum (21,000 ka) and mid-Holocene (6,000 ka) periods. This overlap was located between the latitudes of 25.5–23.5°N, where the hybrid populations are distributed (23.5 and 24.5°N). Similar patterns of secondary contact caused by quaternary oscillations have been recorded in other species and resulted in hybridization (Liu et al., 2014; Marques et al., 2016; Cahill et al., 2018). Our results highlight the role of climatic changes in biodiversity and indicate that Quaternary climate change could favor the scenario of hybridization between the endemic yuccas and establishment of hybrid descendants.

Hybridization over multiple generations causes gradual changes, or clines, in allele frequencies over geographical locations, and a balance between gene flow and natural selection maintains these changes (Barton and Hewitt, 1985). Those loci that display large differences in allele frequencies between parental species can be under strong selection, or linked to selected loci (Yeaman and Otto, 2011). Moreover, these differences can be caused by genetic drift. Among our 3,423 loci, only 174 (5%) fitted the cline model, and 30 of those had a concordant cline center as the hybrid index cline. The displacement of the cline center of the other 144 SNPs suggests asymmetrical gene flow from one of the parental species due to geographical proximity, synchronic flowering, or higher environmental affinities. This displacement also can result from selection against parental variants or allele combinations that do not fit well with the local conditions of the hybrid zone. The other 95% of loci show high variance in allele frequencies across the geographic sampling area, and they could result of the retention of ancestral polymorphism. Our cline analysis confirms the geographic area where hybrids are occurring. The allele frequencies behavior detected provide support, as well done by our ABC models, that these populations are result of admixture from parental species, and they are not a different genetic pool. In the future, when yucca genomes will be available, we will map these candidate loci and associate with morphological features under natural selection. Our results suggest semi-permeable boundaries between genomes (Wu, 2001), which allow some loci to freely introgress and not others. This scenario is common in the early and intermediate stages of divergence among taxa (Baldassarre et al., 2014; De La Torre et al., 2015).

The level of differentiation between parental and hybrid species is influenced by the gene flow rates, and the semi-permeable boundaries between genomes results in differential introgression (Wu, 2001). On a wider scale, the F_ST_ estimations indicate the average level of gene flow between populations. In the current landscape of the peninsula, the geographic distribution of hybrid populations is closer to that of *Y. capensis* than that of *Y. valida*, and it is plausible that higher levels of gene flow exist between hybrids and *Y. capensis* than between hybrids and *Y. valida*. However, the genetic differentiation between the hybrids and both parental species is similar, suggesting similar gene flow rates in both directions. Spatial barriers can limit the gene flow between *Y. valida* and hybrids because there are several hundred kilometers between the geographical ranges of both taxa. In the other hand, temporal barriers can maintain low rates of gene flow between *Y. capensis* and hybrids because the hybrid plants flower in July and August (personal observation) and *Y. capensis* flowers mainly in September and October (Lenz, 1998).

### Environmental Patterns

Reproductive isolation of the hybrid and parental species is an important factor in the speciation process (Schumer et al., 2014). Geographic isolation and ecological divergence can serve as prezygotic barriers. In our study area, yucca hybrid populations occur in areas where parental species are not currently present, which leads to a major geographical isolation barrier to gene flow between parental and hybrid individuals. In addition, we found that most of the environmental axes between the parents and hybrid populations are divergent, achieving our third research goal by indicating that the climatic niche of hybrid populations is shifting from that of their progenitors. Hybrid populations can colonize new ecological spaces that are not utilized by the parental taxa. This ecological divergence could result from a new combinations of traits generated by hybridization, or it can be achieved after speciation through the gradual accumulation of new mutations (Gross and Rieseberg, 2004). Further experiments need to be carried to test which of the hypotheses explains the detected pattern (e.g., Brochmann et al., 2000).

Regions with high environmental heterogeneity favor speciation. The endemic species *Y. valida* occurs mainly in the Vizcaino Desert, and the southern populations of this species are located in the northern part of the Magdalena flatland, while the hybrid populations are distributed in the southern part of the flatland. The climatic conditions of both regions (the Magdalena flatland and Vizcaino Desert) are influenced by the California Current and the associated cold surges (González-Abraham et al., 2010). The habitat of *Y. valida* is extreme in terms of some environmental conditions, exhibiting, for example, the lowest temperatures and low levels of rain, which can explain the partial divergence in climatic niches observed between the taxa. On the other hand, *Y. capensis* is endemic to the Cape Region of Baja California Sur, and it is distributed in the undergrowth of the lowland deciduous forest in the mountains, up to 1,000 meters above sea level (Lenz, 1998; De la Luz et al., 2012). The habitat of *Y. capensis* is much more seasonal than the habitat of hybrid populations, supporting the niche divergence between the taxa.

Geographically explicit predictions of climatic niches are a good starting point to explore niche differences and discover regions with suitable climatic conditions for species. Our SDMs for the present accurately described the distribution of the two endemic species. For the hybrid populations, the model revealed a wide area with suitable conditions that extends beyond the regions where we recorded hybrid individuals. In addition, projecting the models to different times allowed us to link current genomic patterns with historical distributions. For example, *Y. capensis* shows the highest genomic variability, although its geographical range is currently the most restricted. Nevertheless, the predicted distribution of this species during the Last Interglacial and Last Glacial Maximum periods was wider than in the present, suggesting that, in the past, there was a larger suitable area that could support a higher effective population size. On the other hand, *Y. valida*, the parental species with wider distribution, had a low level of genetic diversity and a high number of fixed alleles. This is explained in part by a reduced suitable habitat during the Last Interglacial period that could result in a small effective historical population for this species.

This study confirms how the geographical context influences the specificity of the pollination mutualism between yuccas and yucca-moths. We showed that the hybrid populations in the Baja California Peninsula are the result of combination of the genetic components of the two endemic yucca species. Currently, hybrid individuals with this novel genomic combination occur in different habitats than their parental species, and ecological divergence between them, as well as the spatial and temporal barriers, contributes to reproductive isolation. Finally, to consider these populations as homoploid hybrid species, it is necessary to collect new evidence, which may be related to the role of natural selection in maintaining the distinctions of hybrid taxon as well as the mechanisms through which hybridization generates reproductive isolation (Schumer et al., 2014).

## Data Availability Statement

Raw reads are available in NCBI under the SRA numbers: SRR11514779, SRR11514778, and SRR11514777.

## Author Contributions

All work described in this manuscript was original research carried out by the authors. MA and RB-B developed the original research design and field data collection. JG-P and MA carried out the analyses. All authors contributed substantially to the writing and proofreading of the article, and read and approved the final submitted version.

## Conflict of Interest

The authors declare that the research was conducted in the absence of any commercial or financial relationships that could be construed as a potential conflict of interest.

## Footnotes

1 https://sourceforge.net/projects/bbmap/files/

2 http://worldclim.org/bioclim

## Supplementary Material

FIGURE S1 Models tested for the origin of putative hybrid populations using Approximate Bayesian Computation (ABC) toolbox. Scenario **(A)** ancestral divergence of *Y. valida*, and *Y. capensis* and the posterior origin of the hybrid populations from *Y. valida*; Scenario **(B)** ancestral divergence of *Y. valida* and *Y. capensis*, and the posterior origin of the putative hybrid populations from *Y. capensis*; Scenario **(C)** the divergence of the hybrid populations predates the divergence of *Y. valida* and *Y. capensis*; Scenario **(D)** ancestral divergence of *Y. valida* and *Y. capensis* and the posterior origin of the putative hybrid populations as a result of the admixture of the two genetic pools; Scenario **(E)** the three taxa diverged simultaneously in the past, and they have independent demographic histories.

FIGURE S2 Barplots of the proportion of polymorphic loci for *Y. valida*, *Y. capensis*, and the hybrid populations.

FIGURE S3 Plot of the results of discriminant principal component analysis (DPCA; *K* = 3) for the localities of *Y. valida* (blue), *Y. capensis* (purple), and the hybrid populations (orange). Dots represent individuals.
