# Supplementary figures and images for: Hybridization Between Yuccas From Baja California: Genomic and Environmental Patterns
Source: Front Plant Sci. 2020 May 28;11:685. doi: 10.3389/fpls.2020.00685 (PMC7358647; doi:10.3389/fpls.2020.00685)

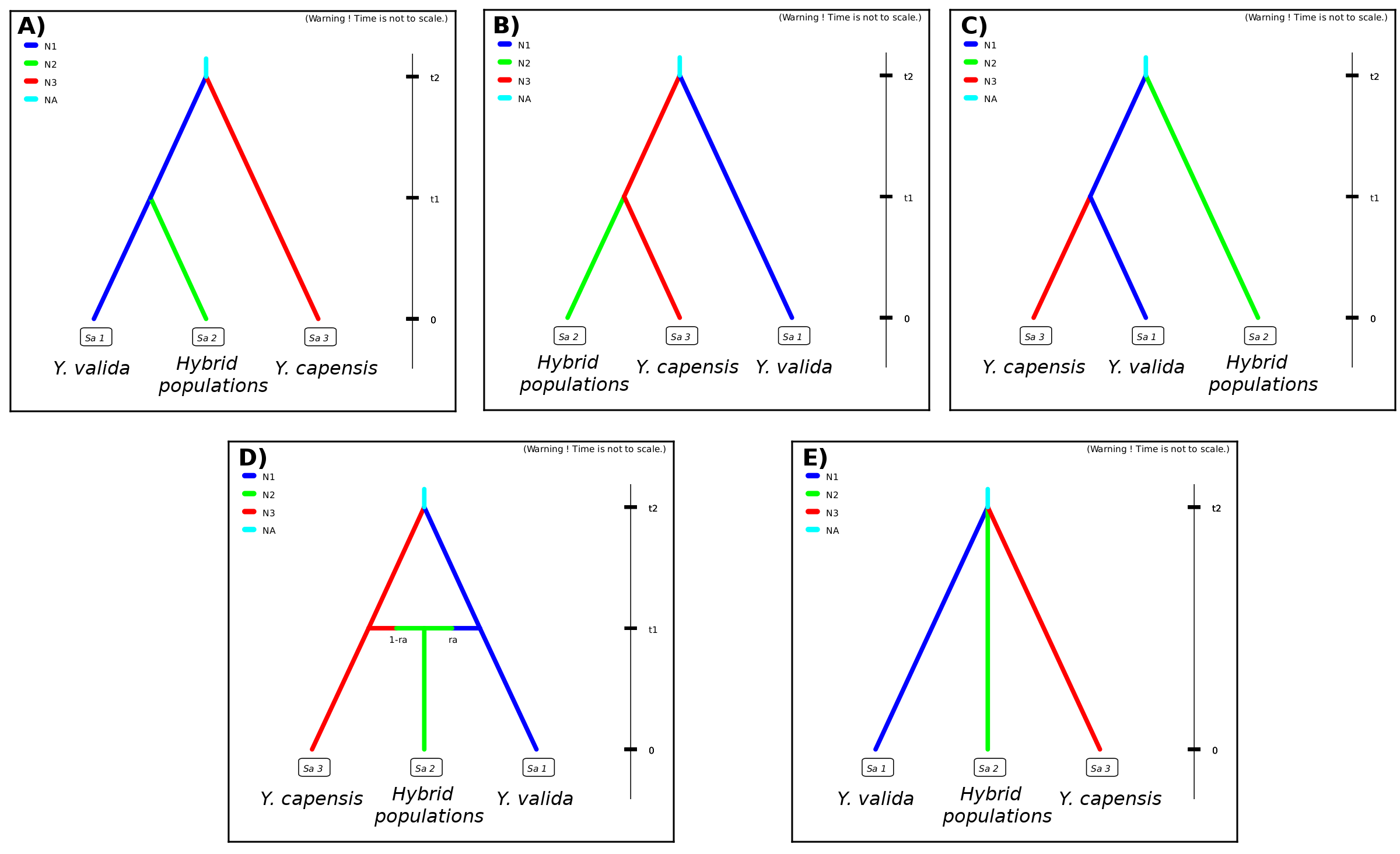

Supplement: FIGURE S1 — Models tested for the origin of putative hybrid populations using Approximate Bayesian Computation (ABC) toolbox. Scenario (A) ancestral divergence of Y. valida, and Y. capensis and the posterior origin of the hybrid populations from Y. valida; Scenario (B) ancestral divergence of Y. valida and Y. capensis, and the posterior origin of the putative hybrid populations from Y. capensis; Scenario (C) the divergence of the hybrid populations predates the divergence of Y. valida and Y. capensis; Scenario (D) ancestral divergence of Y. valida and Y. capensis and the posterior origin of the putative hybrid populations as a result of the admixture of the two genetic pools; Scenario (E) the three taxa diverged simultaneously in the past, and they have independent demographic histories. [file Image_1.PNG]

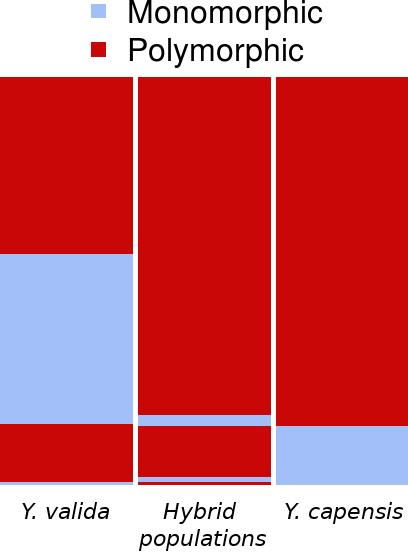

Supplement: FIGURE S2 — Barplots of the proportion of polymorphic loci for Y. valida, Y. capensis, and the hybrid populations. [file Image_2.PNG]

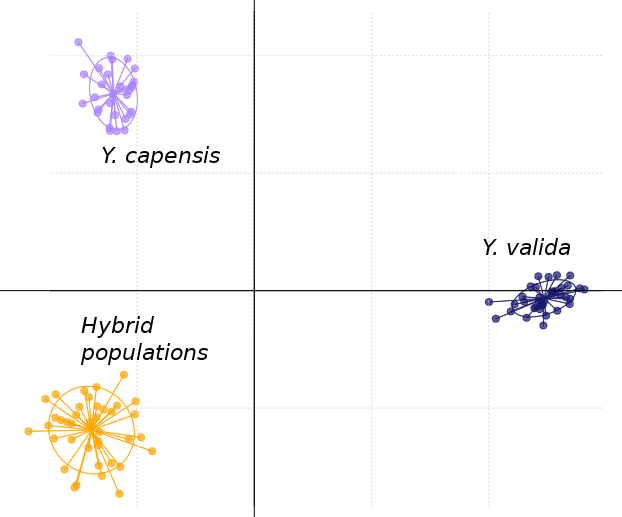

Supplement: FIGURE S3 — Plot of the results of discriminant principal component analysis (DPCA; K = 3) for the localities of Y. valida (blue), Y. capensis (purple), and the hybrid populations (orange). Dots represent individuals. [file Image_3.PNG]
